# Stabilin-2 mediated apoptotic cell phagocytosis induces interleukin-10 expression by p38 and Pbx1 signaling

**DOI:** 10.1007/s12013-024-01243-7

**Published:** 2024-03-13

**Authors:** Han-Seul Jo, Ha-Jeong Kim

**Affiliations:** Department of Physiology, Cell and Matrix Research Institute, Kyungpook National University, School of Medicine, Daegu, South Korea

**Keywords:** Stabilin-2, IL-10, Apoptotic cell phagocytosis, p38 MAPK, Pbx1

## Abstract

Apoptotic cell death occurs under normal physiological conditions, such as development, tissue remodeling, and inflammation. Appropriate removal of apoptotic cells by phagocytes and the secretion of anti-inflammatory cytokines such as IL-10 are important mechanisms for maintaining tissue homeostasis. Apoptotic cell phagocytosis is mediated by several phosphatidylserine recognition receptors on non-professional or professional phagocytes, such as neighboring epithelial cells or macrophages. Stabilin-2 is reported as a phosphatidylserine recognition receptor for apoptotic cell phagocytosis, and its downstream signaling pathway for cytoskeletal rearrangement for phagocytosis is well known. However, the mechanisms for stabilin-2-mediated IL-10 production has not yet been reported. In this study, we aimed to investigate stabilin-2 receptor-mediated IL-10 transcription regulation signaling pathway.

## Highlights

- Stabilin-2 receptor mediated phagocytosis promotes IL-10 production.
- p38 and Pbx1 signaling is mediated to induce IL-10 production.

## Introduction

In multicellular organisms, cells undergo apoptosis during development, tissue remodeling, and inflammation. The rapid clearance of apoptotic cells by phagocytosis is important for maintaining homeostasis [1]. Apoptotic cell clearance by professional and non-professional phagocytes starts with the “recognition of apoptotic cells.” This clearance mechanism is mediated by a complex and well-orchestrated process of interactions among special phagocytic receptors and cellular molecules, as well known as “find-me” and “eat-me” signals [2].

Macrophages, which are professional phagocytes, have several phagocytic receptors that interact with apoptotic cells. These include complement receptors, integrins (αvβ3 and αvβ5), scavenger receptors (SRA, CD36, CD14, and LOX-1), and a phosphatidylserine receptor (stabilin-2) [2]. Furthmore, apoptotic cell triggered TGF-β suppresses LPS-triggered TNF-α release in macrophages [3]. Both release anti-inflamatory cytokines and suppression of pro-inflammatory cytokines are required to prevent the harmful effect in chronic inflammation and the development of autoimmune disorders [2].

Interleukin-10 is produced by phagocytic macrophages and suppresses the activity of antigen-presenting cells and effector lymphocytes. Inappropriate IL-10 expression and its dysregulated activity can cause the generation of self-reactive lymphocytes. Thus, apoptotic cell-induced IL-10 production from phagocytes is important for tissue homeostasis without unwanted inflammation [4–6].

Stabilin-2 is a type I transmembrane protein. Its extracellular region is composed of four clusters containing epidermal growth factor (EGF)-like domain repeats and a fasciclin1 domain. EGF-like domain repeats bind to phosphatidylserine, thus, they are reported as the phosphatidylserine recognition receptors on macrophages [7]. During stabilin-2-mediated phagocytosis, the GULP1, thymosin beta4, arhGAP12, and rac1 pathways are activated for cytoskeletal rearrangement [8–10]. During stabilin-2-mediated apoptotic cell phagocytosis, macrophage release of IL-10 and TGF-β [11]. However, their signal transduction mechanisms have not yet been clarified. In this report, we elucidate the molecular mechanisms involved in the regulation of IL-10 gene expression in stabilin-2-mediated apoptotic cell phagocytosis.

## Materials and method

### Cell culture

THP1 human monocytic cells were purchased from the Korean Collection for Type Cultures. THP1 cells were incubated for 48 h in the presence of 100 ng/ml phorbol 12-myristate-13-acetate (PMA) to induce differentiation. The human stabilin-2-overexpressing cell line, its control L/Mock cells [11], and Jurkat cells were kindly provided by In-San Kim of Kyungpook National University, School of Medicine. The Amaxa 4D Nucleofector X kit (Lonza, Basel, Switzerland) was used for siRNA transfection to THP1 cells following the manufacturer’s protocol.

### Reagents and antibodies

Staurosprine, S203580, and SB202747 were purchased from Calbiochem. Recombinant human IL-10, antibody against stabilin-2 (AF3645), and goat IgG-FITC for flow cytometry were obtained from R&D systems, and antibodies against total phosphorylated p-38, ERK, phosphorylated ERK, Iκ-B, phosphorylated Iκ-B, NF-κB, phosphorylated NF-κB, and β-actin were from Cell Signaling Technology. CMFDA, human stabilin-2 siRNA, human Pbx-1 siRNA, and the control siRNA were purchased from Thermo Fisher Scientific.

### Phagocytosis assay

Apoptotic Jurkat cells were labeled with CMFDA by following the manufacturer’s protocol. Apoptosis was induced with 1 μM staurosporine, a protein kinase C (PKC) inhibitor, for 5 h. One hundred thousand PMA-activated THP1 cells were incubated with 1 × 10^6^ CMFDA-labeled apoptotic Jurkat cells for 1 h at 37 °C. Cells were collected by centrifugation at 100 g to remove apoptotic cells those did not undergo phagocytosis. To quench membrane-bounded apoptotic cell, cells were washed with 0.2% trypan blue. Apoptotic cells were analyzed using Attune Nxt flow cytometry and Attune Nxt software (Life Technologies).

### Flow cytometry assay

Human stabilin-2 expression of the cells were analyzed using an Attune Nxt flow cytometry and Attune Nxt software (Life Technologies). Single cells were suspended with an Fc blocker (BD Pharmingen) for 15 min. Anti-stabilin-2 antibody was added for staining, and was incubated with cells for 1 h at 4 °C. Then, anti-goat IgG-FITC antibody was used to detect the stabilin-2 antibody.

### Immunoblot assay

Cell lysates were obtained using the cell lysis buffer from Cell Signaling Technology, following the manufacturer’s protocol. Cell lysates were resolved by SDS-PAGE, blotted onto an NC membrane, and detected using the indicated antibodies.

### Luciferase assay

The human IL-10 promoter-luciferase construct pIL-10(−1044/ + 30)-luc was generously provided by Xiaojing Ma of Weill Cornel Medical School [12]. The assay was performed using the Dual-Luciferase Reporter Assay system from Promega following the manufacturer’s protocol. Transfection was performed using Lipofectamine 3000 (Thermo Fisher Scientific) following the manufacturer’s protocol.

### Quantitative real-time PCR

Total RNA was extracted using an RNA extraction kit from Takara Bio. Reverse transcription was performed using a cDNA synthesis kit from Takara Bio. Real-time PCR was performed using SYBR Premix Ex Taq from Takara Bio with primer pairs specific for IL-10 (5′-GAGATGCCTTCAGCAGAGTGAAGA-3′, 5′-AGTTCACATGCGCCTTGATGTC-3′) or GAPDH (5′-TCACCACCATGGAGAAGGC-3′, 5′-GCTAAGCAGTTGGTGGTGCA-3′). Data were processed as fold changes in mRNA expression relative to GAPDH expression.

### ELISA

The concentration of interleukin-10 (IL-10) in the culture supernatant was measured using an enzyme-linked immunosorbent assay (ELISA) kit (R&D System) according to the manufacturer’s protocol.

### Statistical analysis

The results are expressed as the mean ± S.D. from at least three independent experiments. All statistical analyses were performed using the unpaired *t* test. Data were considered significant if *p* < 0.05.

## Results

### Stabilin-2 mediates apoptotic cell phagocytosis

**Fig. 1:**
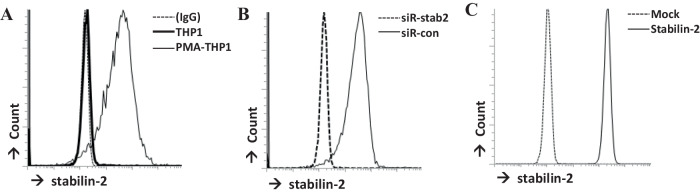
Stabilin-2 expression. **A** in THP1 cells and PMA-treated THP1cells (PMA-THP1) **B** THP1 cells were transfected with control siRNA (siR-con) or stabilin-2 siRNA (siR-stab2). **C** mouse fibroblastic L cells were transfected with a pcDNA expression vector (Mock) or stabilin-2 expression construct (stabilin-2). Stabilin-2 expression was detected by flow cytometry, and each of the cells were stained anti-stabilin-2 antibody

**Fig. 2:**
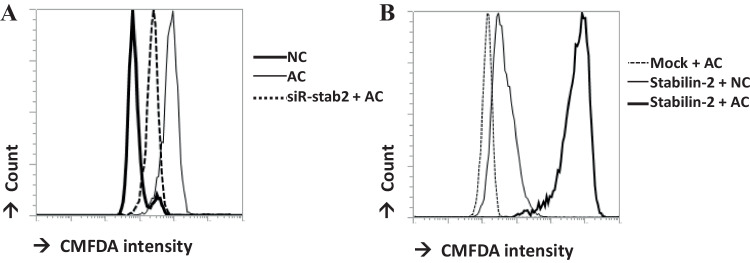
Phagocytosis assay. CMFDA-labeled apoptotic Jurkat cells were prepared as described. **A** PMA-treated THP1 cells were incubated with normal Jurkat cells (NC) or apoptotic Jurkat cells (AC). siRNA-transfected THP1 (siR-stab2) cells were also incubated with apoptotic Jurkat cells (AC). **B** pcDNA expression vector-transfected L cells (Mock) or stabilin-2 expression construct-transfected stable L cells (stabilin-2) were incubated with normal Jurkat cells (NC) or apoptotic Jurkat cells (AC). Engulfed CMFDA-labeled apoptotic Jurkat cells were analyzed by flow cytometry

Stabilin-2 expression on the THP1 human monocytic cell line was measured by flow cytometry. It was found that stabilin-2 was expressed on PMA-pretreated THP1 cells, but not in inactivated THP1 cells. (Fig. 1A) To investigate stabilin-2-mediated apoptotic phagocytosis, stabilin-2-deficient THP1 cells were generated using stabilin-2 siRNA. After stabilin-2 siRNA transfection, stabilin-2 was not expressed on the surface of PMA-treated THP1 cells. (Fig. 1B) Apoptotic cell phagocytosis was only found in PMA-treated THP1 cells, but not in inactivated THP1 cells. This apoptotic cell phagocytosis was decreased in stabilin-2-deficient cells. (Fig. 2A).

To establish the role of stabilin-2 in apoptotic cell phagocytosis, a stabilin-2-overexpressing cell line was used [11]. Human stabilin-2 expression was confirmed in stably transfected cells, but not in non-transfected mouse fibroblastic L cells (Mock). (Fig. 1C) Apoptotic cell phagocytosis was found only in cells overexpressing stabilin-2, but not in non-transfected cells. This result was similar with previous experiment using aged RBC by Park et al. [11]. Normal cells (NC) were not taken up by stabilin-2-overexpressing cells. (Fig. 2B) These data suggests that stabilin-2 is important for apoptotic cell phagocytosis.

### Stabilin-2 mediated apoptotic cell phagocytosis induces IL-10 production

**Fig. 3:**
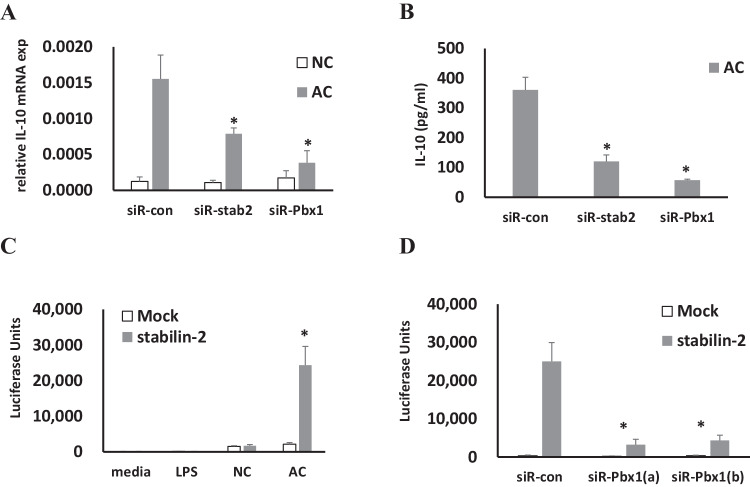
IL-10 expression analysis. **A** IL-10 mRNA expression was performed by q-PCR as mentioned materials and methods. Stabilin-2 siRNA (siR-satab2) or Pbx-1 siRNA (siR-Pbx1) were transfected to THP1 cells, and were subjected to PMA treatment for 48 h. CMFDA labeled with normal Jurkat cells (NC) or apoptotic Jurkat cells (AC) were incubated with THP1 cells. **B** IL-10 protein was measured by ELISA. *p < 0.05 compared with siR-con group. **C** IL-10 promoter-luc construct was transfected to pcDNA expression vector transfected L cells (Mock) or stabilin-2 expression construct transfected stable L cells (stabilin-2). LPS, normal Jurkat cells (NC), and apoptotic Jurkat cells (AC) were incubated with Mock or stabilin-2 cells. Luciferase assay was performed as mentioned in materials and methods. *p < 0.05 com*p*ared with NC group. **D** IL-10 promoter-luc construct and stabilin-2 siRNA (siR-satab2) or Pbx-1 siRNA (siR-Pbx1) was transfected to pcDNA expression vector transfected L cells (Mock) or stabilin-2 expression construct transfected stable L cells (stabilin-2). Apoptotic Jurkat cells were incubated with cells and luciferase assay was performed as mentioned in materials and methods. All data are presented as mean ± SD from a minimum of three individual experiments. *p < 0.05 compaired with siR-con group

Apoptotic cells induced IL-10 mRNA and protein expression, but not normal cells (NC). Apoptotic cell-induced IL-10 expression was decreased in cells deficient in stabilin-2. IL-10 mRNA and protein production also decreased in macrophages that do not express Pbx-1, an IL-10 transcription regulator. (Fig. 3A, B).

To confirm stabilin-2-specific regulation of IL-10 transcription, an IL-10 promoter-reporter was used. Constructs of the −1042/ + 30 IL-10 promoter were transfected into stabilin-2-expressing or non-expressing cells, and the reporter activity was measured after apoptotic cell treatment. Apoptotic cell-induced IL-10 promoter activity was only found in stabilin-2-expressing cells, but not in stabilin-2-non-expressing cells. LPS and normal cells did not induce IL-10 promoter activity. (Fig. 3C) This apoptotic cell-induced IL-10 promoter activity was not found in Pbx-1-deficient cells. (Fig. 3D) These data suggest that the stabilin-2 receptor signal by apoptotic cells was involved in IL-10 transcription regulation.

### Stabilin-2 receptor induces p38 MAPK activation by apoptotic cell phagocytosis

**Fig. 4:**
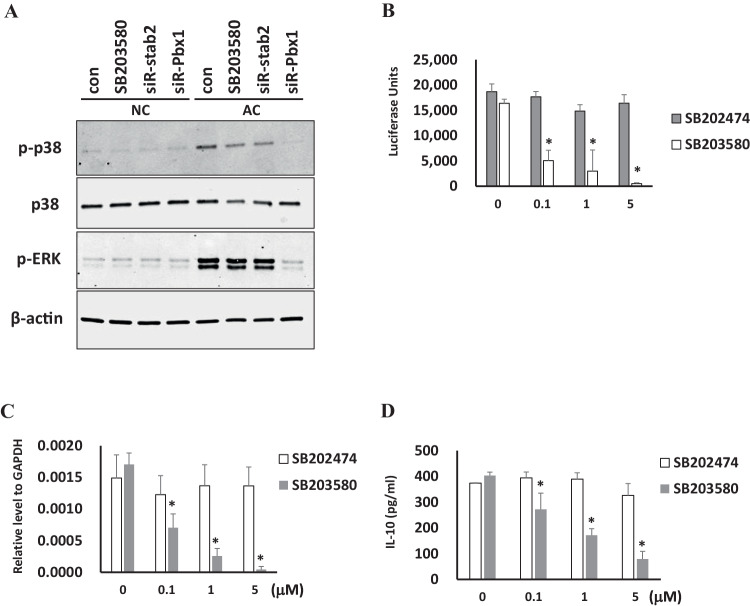
p38 MAPK is important in stabilin-2 mediated apoptotic cell-induced IL-10 promoter and protein production. **A** Stabilin-2 siRNA (siR-satab2) or Pbx-1 siRNA (siR-Pbx1) were transfected to THP1 cells. PMA-activated THP1 cells were incubated with normal Jurkat cells (NC) or apoptotic Jurkat cells (AC). Cells were collected and the lysates were collected with a lysis buffer. Phosphor-p38, p-38, phosphor-ERK, and ERK protein were detected by immunoblots. **B** An IL-10 promoter-luc construct was transfected to stabilin-2 expression construct-transfected stable L cells (stabilin-2). Apoptotic Jurkat cells were incubated with the indicated concentrations of the inhibitor, and the luciferase assay was performed as mentioned in the Materials and Methods. **C, D** PMA-activated THP1 cells were incubated with the indicated concentration of inhibitor and apoptotic Jurkat cells, and the mRNA and protein were collected, where q-PCR and ELISA were performed, respectively. All data are presented as mean ± S.D. from a minimum of three individual experiments. *p < 0.05 compared with siR-con group

It has been shown that apoptotic cells induce p38 MAPK phosphorylation, since normal cells do not induce p38 MAPK phosphorylation [12]. This apoptotic cell-induced p38 MAPK phosphorylation decreased after SB203580 treatment, an inhibitor of p38 MAPK. In stabilin-2-deficient cells, p38 MAPK phosphorylation was decreased after apoptotic cell treatment. (Fig. 4A) Apoptotic cells also induced ERK phosphorylation, but this phosphorylation was not inhibited by SB203580 and stabilin-2 siRNA. These data indicate that apoptotic cell-induced p38 MAPK signaling was regulated by a stabilin-2 receptor. (Fig. 4B)

To determine the relationship between p38 MAPK signaling and IL-10 transcription regulation, we examined IL-10 promoter activity after treatment with SB203580, an inhibitor of p38 MAPK. As shown in Fig. 3B, SB203580 inhibited apoptotic cell-induced IL-10 promoter reporter activity, but not SB202374, a control inhibitor. IL-10 mRNA and protein production was also inhibited by SB203580 in a dose-dependent manner. (Fig. 4C, D).

### Stabilin-2 mediated apoptotic cell phagocytosis suppressed pro-inflammatory signaling

**Fig. 5:**
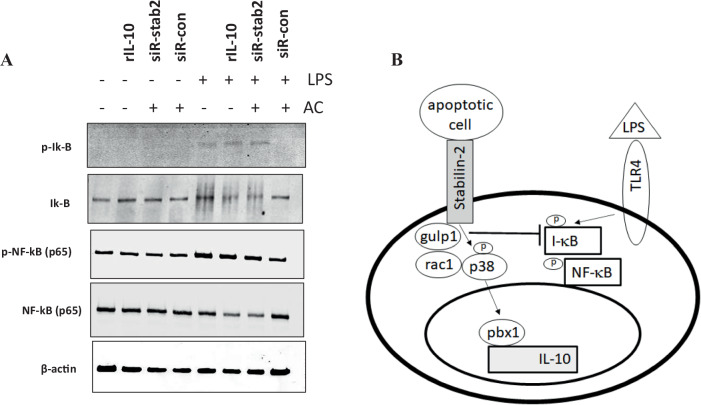
Stabilin-2-mediated apoptotic cell phagocytosis suppresses the pro-inflammatory response. **A** PMA-activated THP1 cells were incubated with the indicated materials and the cell lysates were collected. Immunoblotting was performed using the indicated antibodies. **B** Model for IL-10 expression of p38 and pbx1 signaling by stabilin-2-mediated apoptotic cell phagocytosis

It is well known that apoptotic cell phagocytosis suppresses pro-inflammatory responses, such as IkB and p65 NF-kB signaling [2]. As shown is Fig. 5, LPS induced IkB and p65 NF-kB phosphorylation, but apoptotic cell itself did not. LPS stimulated IkB and p65 NF-kB phosphorylation was inhibited by apoptotic cell treatment. This inhibitory effect by apoptotic cell was disappeared in stabilin-2-deficient cells. Recombinant IL-10 did not suppress LPS induced IkB and p65 NF-kB phosphorylation. (Fig. 5) These data suggest that stabilin-2 receptor stimulation by apoptotic cell suppressed pro-inflammatory signal, but it is not IL-10 dependent.

## Discussion

Stabilin-2 is the receptor for the recognition of phosphatidylserine (PS) in apoptotic cells. It is found on macrophages and it mediates the phagocytosis of apoptotic cells [7, 11]. We also confirm that mouse fibroblastic L cells without stabilin-2 expression cannot engulf apoptotic cells, however stabilin-2 overexpressed L cell engulf apoptotic cells. (Fig. 2B) (because this is same as previous sentence.) In our data, stabilin-2 was not found on THP1 monocytic cells, but it was found on PMA-activated THP1 cells. (Fig. 1A) This result means stabilin-2 can be found at least on macrophages, even though its expression on monocytes is not yet clear. We also showed the apoptotic cell phagocytic capacity of stabilin-2. The phagocytic capacity of THP1 cells decreased when stabilin-2 expression was decreased by siRNA-stabilin-2 (Fig. 1B, C), although the phagocytic capacity of THP1 remained in stabilin-2-deficient THP1 cells. These data indicate that stabilin-2 is the receptor for apoptotic cell phagocytosis on macrophages, but other receptors exist as well, such as integrins (αvβ3, αvβ5), CD36, and LOX-1 [2].

Apoptotic cell clearance is a multistep process, and its impairment can cause autoimmune diseases. Both recognition and cellular responses should be well-regulated [1]. Alterations in cellular responses are critical for the immune phenotype. Impaired responses can result in inflammation-associated pathologies or autoimmunity, which are generated by self-reactive lymphocytes [2]. IL-10 plays a central role in restricting inflammatory responses [6]. It was discovered as a product of Th2 cells that inhibit Th1 cytokine production [13]. IL-10 deficient mice show a stronger phenotype in several animal inflammatory models, such as chronic enterocolitis, endotoxin shock, and autoimmune encephalitis [5, 14, 15]. Serum levels of IL-10 were increased in several chronic inflammatory diseases [16]. Many receptors, which mediate the recognition and phagocytosis of apoptotic cells, are implicated in IL-10 production [2, 3, 11, 12, 17, 18]. In this report, we also show that stabilin-2 mediated apoptotic cell phagocytosis triggers IL-10 gene expression. In THP1 cells, IL-10 gene expression and protein secretion triggered by apoptotic cell decreased in stabilin-2 deficient cells. (Fig. 2A, B) IL-10 promoter reporter activity was found in stabilin-2 expressing cells, but not in non-expressing cells. (Fig. 2C) Apoptotic cells and IL-10 cannot induce phosphorylation of IκB and NF-κB, which are the main regulators for proinflammatory responses. However, apoptotic cells suppressed LPS-induced phosphorylation of IκB and NF-κB. (Fig. 4).

IL-10 gene expression in macrophages is usually induced by several inflammatory triggers, such as LPS. However, the kinetics of IL-10 differs from other typical inflammatory cytokines [19, 20]. LPS-induced IL-10 production is dependent on the signaling cascade of p38, not p42 (also called extracellular signal regulated kinase 1, ERK1), and mitogen-activated protein kinase (MAPK) [21]. However, apoptotic cell induced IL-10 production is also dependent on p38 signaling [12]. We also confirmed p38 phosphorylation after apoptotic cell phagocytosis. This p38 phosphorylation decreased in stabilin-2 deficient THP1 cells. (Fig. 3A) The specific p38 inhibitor, SB203580, suppressed apoptotic cell induced IL-10 promoter activity in stabilin-2 expressed L cells. (Fig. 3B) IL-10 mRNA expression and protein secretion were also decreased by SB203580 in a dose dependent manner (Fig. 3C, D).

Pbx1 is reported to be a transcription factor for IL-10 gene expression following apoptotic cell phagocytosis [12]. Apoptotic cells induced IL-10 mRNA and protein levels to decrease in Pbx1 deficient cells. (Fig. 2A, B) IL-10 promoter activity was also not found in Pbx1 deficient cells. (Fig. 2C, D) These data suggest that Pbx1 is required in stabilin-2 mediated IL-10 production.

## Conclusions

In this report, we showed that apoptotic cell phagocytosis is mediated by the stabilin-2 receptor on macrophages. Stabilin-2 activation by apoptotic cells triggers IL-10 generation, which requires the p38 MAPK signaling pathway and Pbx1. Apoptotic cells induced stabilin-2 activation suppresses LPS induced inflammatory response. Based on these data, an impaired immune response might occur when a stabilin-2 receptor does not work.

### Supplementary Information

Supplementary Information

## Data Availability

No datasets were generated or analysed during the current study.
