# Supplementary figures and images for: Stabilin-2 mediated apoptotic cell phagocytosis induces interleukin-10 expression by p38 and Pbx1 signaling
Source: Cell Biochem Biophys. 2024 Mar 13;82(2):919–25. doi: 10.1007/s12013-024-01243-7 (PMC11344723; doi:10.1007/s12013-024-01243-7)

Supplement Figure 1.

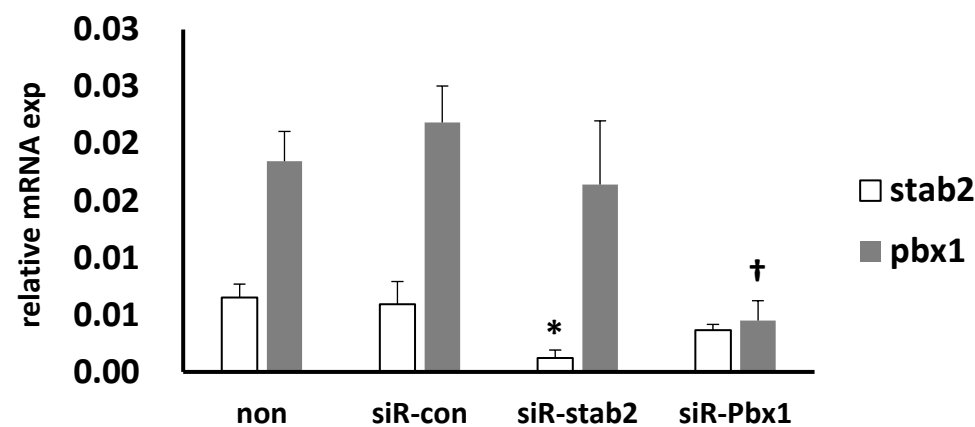

Supplement: Supplementary file 1 — Supplementary Information [file 12013_2024_1243_MOESM1_ESM.pdf]
